# Valorization of Yellowfin Tuna Tails: From Proteolytic Enzyme Production to Gelatin and Antioxidant Hydrolysate Extraction

**DOI:** 10.3390/foods13132034

**Published:** 2024-06-27

**Authors:** Alisson Sisa, Oscar Martínez-Álvarez, Joaquín Gómez-Estaca, Mauricio Mosquera

**Affiliations:** 1 Department of Food Science and Biotechnology (DECAB), Escuela Politécnica Nacional, Quito P.O. Box 17-01-2759, Ecuador; 2 Institute of Food Science, Technology and Nutrition (ICTAN-CSIC), 6th José Antonio Novais St., 28040 Madrid, Spain

**Keywords:** yellowfin tuna (*Thunnus albacares*), valorization, by-products, proteolytic enzymes, gelatin, antioxidant peptides

## Abstract

This study investigates the valorization potential of yellowfin tuna (*Thunnus albacares*) tails to produce high-value commercial products. Firstly, the tuna tails were placed in a perforated stainless-steel cylinder, and hydraulic pressure was applied to separate the skin from the muscle in the tails. The extracted muscle was then utilized as a nitrogen source for the growth of the proteolytic enzyme producer *Bacillus subtilis*, while the skins were employed for gelatin extraction. The proteases from *B. subtilis* were partially purified and used to produce antioxidant peptides from the obtained gelatin. The gelatin formed a gel upon cooling, with gelling and melting temperatures of 16 °C and 22 °C, respectively, and a Bloom strength of approximately 160. Response Surface Methodology (RSM) was employed to determine the optimal hydrolysis conditions to achieve the highest antioxidant activity (35.96% measured as DPPH radical scavenging activity), which were 50 °C and 6.5 IU of enzyme. The findings emphasize the importance of an integrated approach to maximize the value of tuna by-products, promoting sustainability within the framework of a circular bioeconomy. Overall, these results contribute to the efficient utilization of tuna by-products, waste reduction, and enhanced economic viability of the tuna industry.

## 1. Introduction

The tuna industry holds great global significance, providing substantial employment opportunities and making significant contributions to the global economy [1]. Global catches of tunas and related species (including *Thunnus albacares*, *Thunnus maccoyii*, *Thunnus obesus*, *Thunnus thynnus*, *Thunnus alalunga*, and *Katsuwonus pelamis*) reached a volume of 7.8 million tons in 2020 [2]. The industrial processing of tuna generates a substantial quantity of solid waste, including muscle trimmings, viscera, gills, dark muscle, heads, tails, bones, skins, and fins, which constitutes up to 70% of the initial raw material [3]. Although these by-products are useful in the production of fertilizers or fishmeal/fish oil for aquaculture fish feed or pet food, there is untapped potential to create higher value products due to their protein-rich composition, thereby enhancing the economic sustainability of this industry [4]. However, a stark reality persists that significant quantities of these by-products are discarded, resulting in considerable environmental damage [5]. Consequently, there is an urgent need to develop strategies for the valorization of these by-products, with a primary focus on generating high-value products. One interesting approach involves converting protein-rich by-products into protein hydrolysates with bioactive and nutritional characteristics. Alternatively, collagen/gelatin can be extracted from the skin for various purposes [6,7,8] and represents a good alternative to mammalian gelatin. It can be utilized as a food ingredient as an emulsifier, to produce edible films, or even for the development of functional foods [9,10].

The utilization of protein-rich by-products as a substrate for protease production is of great interest due to the abundance and underutilization of these resources. It has been demonstrated that several microorganisms are able to utilize these by-products as a source of carbon, nitrogen, and energy in the enzyme production process, which has generated scientific and industrial interest. This interest stems from the fact that nearly 50% of the cost associated with enzyme production is attributed to the raw material, namely the substrate for the growth of enzyme-producing microorganisms [11]. Microorganisms secrete proteolytic enzymes into the culture medium during the growth phase that have excellent properties for application in hydrolysis processes [12]. This strategic approach presents a promising avenue for the efficient production of proteolytic enzymes with industrial relevance, leveraging economically accessible resources and contributing to the valorization of by-products [13]. Proteases of microbial origin offer distinct advantages over those of plant and animal origin, including ease of large-scale production, rapid growth, and lower production costs, among others [14].

Bioactive peptides are present within the sequence of native proteins and must be released by hydrolysis to exert their biological functions. Among the array of hydrolysis methods available, utilizing proteases from animal, plant, or microbial sources is feasible. However, the use of microbial proteases is preferable due to their rapid reaction and enhanced stability [15]. In comparison to chemical hydrolysis, enzymatic hydrolysis offers several advantages. These include the preservation of the nutritional value, the absence of residual organic or chemical solvents, and its rapid, safe, and easily regulated nature [16,17].

Protein hydrolysates consist of free amino acids and peptides with varying molecular weights that exhibit distinct technological and functional properties. Due to their smaller size, the constituent amino acids are more easily absorbed in the small intestine, fulfilling diverse physiological functions in the human body [18]. Peptides offer a range of health benefits, including the potential treatment of certain diseases. They may also possess specific activities of technological interest, such as antioxidant and antimicrobial properties [19]. Peptides derived from collagen and gelatin have demonstrated numerous bioactivities, including antioxidant, antihypertensive/ACE-inhibitory, antimicrobial, mineral-binding capacity, lipid-reducing effects, immunomodulatory activity, and beneficial effects on skin, bones, and joints [20,21]. Recent studies have highlighted the preparation of functional components using tuna processing by-products. For instance, bioactive peptides from skipjack tuna (*K. pelamis*) cardiac arterial bulbs have exhibited protective functions on UVB-irradiated HaCaT cells through antioxidant and anti-apoptotic mechanisms [22]. Additionally, gelatin and antioxidant peptides derived from the skins of skipjack tuna (*K. pelamis*) have shown significant bioactivity, including radical scavenging and cytoprotective abilities [23].

Oxidative reactions represent a primary cause of food spoilage, resulting in the formation of free radicals and compounds that can cause chronic diseases by damaging cell membranes and biomacromolecules. These diseases include diabetes mellitus, cancer, and liver disease [24]. In recent years, there has been a trend towards utilizing additives of natural origin. As a result, marine sources have garnered attention for the extraction of antioxidant peptides. For instance, the muscle of the Corvina fish (*Miichthys miiuy*) has been hydrolyzed using different enzymes (alcalase, trypsin, papain and pepsin), resulting in the release of 10 peptides with strong antioxidant activity [18]. The viscera of tilapia (*Oreochromis* spp.) have also been employed as a source of antioxidant peptides, with positive results [25]. Furthermore, carp skins have been tested as a raw material for the release of antioxidant peptides by hydrolysis with alcalase [26]. In practical applications, antioxidant peptides have demonstrated effectiveness in various contexts. For instance, they have shown promising results in reducing lipid oxidation in meat [27]. Additionally, incorporating antioxidant peptides into the formulation of flour for biscuit production has led to notable improvements in both the nutritional value and antioxidant capacity of the final product [28].

The primary objective of this study is to valorize yellowfin tuna (*T. albacares*) tails through an integrated approach involving the separate exploitation of different components of the by-product. Initially, the muscle protein was evaluated as a nitrogen source to produce proteases by *B. subtilis*. These proteases were then used to release antioxidant peptides from gelatin extracted from the skin. The evaluation of gelatin quality and the optimization of hydrolysis conditions with the objective to maximize antioxidant activity represent additional objectives of this study. 

## 2. Materials and Methods

### 2.1. Sample Collection and Preparation

The tails of yellowfin tuna (*T. albacares*) were obtained from a local market in Quito, Ecuador. The tails were carefully selected to ensure freshness and promptly transported on ice to the laboratory for further processing. To separate the muscle tissue from the skin, six tails were placed into a specially designed perforated stainless-steel cylinder. This cylinder allowed liquid to pass through while retaining solid components, achieved through low pressure magnified by fluid pressure effects. Hydraulic pressure was applied to the tails using a Sematech Engineering press (Quito, Ecuador), reaching pressures of up to 6000 pounds per square inch (PSI). This method effectively extracted the desired muscle tissue, leaving the skin inside the cylinder. The extracted muscle was subsequently freeze-dried, minced, and stored at −80 °C until further use.

### 2.2. Chemicals and Reagents

All experimental procedures were conducted using analytical-grade reagents, including potassium phosphate monobasic and dibasic, sodium chloride, Tris-HCl (tris(hydroxymethyl)aminomethane hydrochloride), and trichloroacetic acid, obtained from Thermo Fisher Scientific (Waltham, MA, USA). Additionally, BHI–Agar (Brain Heart Infusion with agar), dextrose, azocasein, SDS (sodium dodecyl sulfate), TEMED (N,N,N′,N′-tetramethylethylenediamine), Tricine, Sephadex G-100, and Sephadex G-25 resin were obtained from Sigma Aldrich (St. Louis, MO, USA), while ammonium sulfate, 2-mercaptoethanol, glycerol, sodium hydroxide, and BHI (Brain Heart Infusion) broth were purchased from Merck KGaA (Darmstadt, Germany). Ammonium persulfate, Bromophenol brilliant blue, Coomassie Brilliant Blue, acrylamide, and bisacrylamide were obtained from Bio-Rad (Hercules, CA, USA).

### 2.3. Enzymatic Extract Preparation

The enzymatic extract was obtained by cultivation of Bacillus subtilis in a culture medium prepared with 0.5% sodium chloride, 0.2% dextrose, and 1% freeze-dried yellowfin tuna muscle. The components were dissolved in 0.1 M phosphate buffer (pH 7) and then sterilized (121 °C, 15 min) in a Trident EA-632 autoclave (Taiwan). The strain of B. subtilis was isolated and identified in a previous study [29]. The B. subtilis strain stored at −80 °C in BHI broth was thawed under refrigeration. Then, 100 µL of bacteria was inoculated into BHI broth and incubated at 37 °C for 24 h. It was then inoculated into the broth prepared with yellowfin tuna muscle and incubated for 3 days at 37 °C under aerobic conditions with continuous shaking at 180 rpm. After the incubation period, the enzyme extract was obtained by precipitation with ammonium sulfate. For this, the fermented culture medium was mixed with ammonium sulfate at a concentration of 40% (*w*/*v*) and stirred continuously on ice for 1 h. The mixture was then centrifuged at 5500× *g* for 15 min at 25 °C to separate the precipitate (containing the enzymes) from the supernatant (containing the bacterial cells).

### 2.4. Purification of the Enzymatic Extract

The enzymatic extract obtained after precipitation was desalted using a pre-hydrated Sephadex G-25 gel filtration column (4 mm diameter, 40 cm height) [30], pre-equilibrated in 0.1 M phosphate buffer. The enzymatic extract was then semi-purified using a column packed with Sephadex G-100 resin (7.5 mm diameter, 10 cm height), previously hydrated in 0.1 M phosphate buffer at pH 7 for 24 h [31]. During this process, 15 fractions of 1 mL each were collected under a constant flow of 0.1 M phosphate buffer, at pH 7.

### 2.5. Determination of Proteolytic Activity of Alkaline Proteases

The proteolytic activity of the collected samples was determined using azocasein as a substrate [32]. For that, a reaction mixture was prepared by mixing 200 µL of Tris-HCl buffer (0.1 M, pH 8), 200 µL of the extract, and 200 µL of 1% (*w*/*v*) azocasein. A blank solution was prepared by combining 200 µL of 1% azocasein, 1 mL of 10% trichloroacetic acid (TCA), 200 µL of Tris-HCl buffer, and 200 µL of distilled water. The reaction mixture was then incubated at 37 °C for 30 min. Then, 1 mL of TCA solution was added, followed by centrifugation at 5500× *g* for 15 min. After centrifugation, 400 µL of 1.8 N NaOH was added to the supernatant. The absorbance of the resulting solution was measured at a wavelength of 420 nm using a UV/Vis spectrophotometer (UV-160A, Shimadzu, Kyoto, Japan).

Under the specified measurement conditions, one unit of proteolytic activity (U) was defined as the amount of enzymatic extract (mL) that resulted in a 0.1 increase in absorbance per minute. Azocaseinolytic activity was quantified by expressing the proteolytic activity in units per milliliter (U/mL).

The fraction containing the enzymes was supplemented with 1% glycerol to improve stability and then freeze-dried to obtain a stable form.

### 2.6. Molecular Size Determination

The fraction containing the enzymes was subjected to polyacrylamide gel electrophoresis analysis [33] using 10% acrylamide gels prepared from a 49.5%T, 3%C acrylamide/bisacrylamide mixture (T denotes the total percentage concentration of both monomers (acrylamide and bisacrylamide) and C denotes the percentage concentration of the cross-linking agent relative to the total concentration of T). A constant voltage of 110 V was used to run the samples. To prepare the sample, 20 mg of lyophilized samples was dissolved in 1 mL of a denaturing solution (50 mM Tris, 4% SDS, 2% mercaptoethanol, 12% glycerol, and 0.01% bromophenol blue) adjusted to pH 6.8 with 1 N HCl. The solution was boiled for 10 min and then centrifuged at 10,000× *g*. The protein bands were subsequently stained with Coomassie blue. A molecular weight marker ranging from 6.5 to 200 kDa (Sigma Marker S8445-10VL, Sigma-Aldrich, St. Louis, MO, USA) was used to determine the tentative molecular weight of the proteins in the stained bands.

### 2.7. Gelatin Extraction from Yellowfin Tuna

Firstly, the skins were washed with two volumes of a 5% NaCl solution for 30 min to remove any non-collagenous adhered proteins. The process was repeated twice. Subsequently, the skins were immersed in 0.1 N NaOH solution for one hour to eliminate any remaining lipids. To ensure complete lipid removal, the samples were afterwards treated thrice with two volumes of 10% isobutyl alcohol for 30 min. Finally, the samples were thoroughly washed with distilled water and treated with 0.05 M acetic acid for 21 h at room temperature. The final extraction of gelatin was performed twice with distilled water at 60 °C overnight. The extracts were filtered using gauze, then dried in a forced-air oven at 45 °C for 12 h. The gelatin was finally ground into a fine powder and stored at −20 °C until use [20].

### 2.8. Amino Acid Analysis

Gelatin was dissolved (1 mg/mL) in ultrapure water and further hydrolyzed in a vacuum-sealed glass at 110 °C for 24 h in the presence of continuously boiling 6 N HCl containing 0.1% phenol and norleucine as internal standard. After hydrolysis, the sample was again vacuum-dried, dissolved in application buffer, and injected onto a Biochrom 30 amino acid analyser (Biochrom Ltd., Cambridge, UK) equipped with an LKB Ultropack 8 resin column (Pharmacia LKB Biotechnology, Inc. Pascataway, NJ, USA). The results were expressed as grams per 100 g of amino acids (%).

### 2.9. Gel-Forming Properties

Gelatin (6.67 g/100 mL) was dissolved in distilled water at 40 °C for 20 min. The viscoelastic properties of the gelatin were evaluated using a rotational rheometer (Advanced Rheometer AR 2000, TA Instruments Ltd., Newcastle, UK) equipped with a 2° cone angle and a 40 mm plate distance. A dynamic temperature sweep was performed by subjecting the gelatin to a temperature sweep from 35 to 5 °C and then returning it to 35 °C. The sweep rate was 1 °C per minute, with a frequency of 0.5 Hz, an initial stress of 3 Pa, and a deformation of 0.2 [34].

The gelation and subsequent melting points of the gelatin were evaluated by analyzing the elastic modulus, viscous modulus, and phase angle as a function of temperature. To study the dynamic frequency sweep, the gelatin was maintained at 4 °C, and oscillatory measurements were performed over a frequency range of 0.1 to 10 Hz, with an oscillation amplitude (strain) within the linear range (0.005). The elastic and viscous moduli were measured as a function of frequency, providing valuable insight into the mechanical properties of the gelatin under varying deformation rates.

### 2.10. Gel Strength

The gel strength of the gelatin extract was measured as described by Boran and Regenstein [35] with some modifications, employing a Perten Instruments TVT 6700 texturometer (PerkinElmer Company, Sydney, Australia) equipped with a 40 mm diameter flag compression. Gelatin (6.67%) was dissolved in distilled water at 45 °C, introduced into 100 mL containers measuring 60 mm in height and 50 mm in diameter, and then cooled to 7 °C over a period of 15 h. Gel strength was measured at 7 °C, as determined by the maximum force (in grams) required to penetrate 4 mm of gelatin with the plunger. The results were the average of at least five determinations [34].

### 2.11. Optimization of Gelatin Hydrolysis Conditions

The optimization of gelatin hydrolysis to achieve the optimal degree of hydrolysis was performed using a response surface experimental design. This design included three temperature levels (50 °C, 60 °C, and 70 °C) and three enzyme concentrations, expressed in enzymatic units per gram of dehydrated gelatin (1.5, 4, and 6.5). Two replicates were performed at the central point. For the optimization process, the degree of hydrolysis (DH) was chosen as the response variable. The hydrolysis reaction was initiated by dissolving one gram of dehydrated gelatin in 100 mL of distilled water and adjusting the pH of the mixture to 8. The previously obtained lyophilized enzyme extract was then added to the mixture. These temperature conditions and enzyme concentration settings were based on previous studies that investigated the isolation of B. subtilis and its enzyme production capabilities in different culture media [36], and its application for the hydrolysis of bovine blood [37].

The degree of hydrolysis (DH) was evaluated by the pH-stat method [38]. The amount of NaOH 0.1 N consumed to maintain a pH value of 8 during the protein hydrolysis was recorded at fixed intervals throughout the hydrolysis process. An 848 Titrino plus complete titrator (Metrohm Inc., Tampa, FL, USA) was used to maintain a constant pH and to obtain the NaOH consumption data. Upon completion of the hydrolysis process, the enzyme was inactivated by heating at 95 ± 0.3 °C for 10 min in a thermostatic bath followed by cooling it to room temperature [39]. The results were expressed as the percentage of cleaved peptides (h) relative to the total number of peptide bonds available for proteolysis as a percentage (Equation (1)):

(1) $$DH\;\left(\%\right)=\frac{h}{h_{total}}\times 100=\frac{N_{b}\;\times \;V_{NaOH}}{MP\;\times \;\alpha \;\times h_{total}}\times 100$$

where *V*_*NaOH*_ is the volume of NaOH consumed (mL). *N*_*b*_ is the normality of the base. *MP* is the protein mass (g). *h_total_* was calculated from the amino acid profile and expresses the total number of peptide bonds in gelatin (10.82 mEq/g protein), and ∝ is the dissociation degree of the ∝NH2 groups released during the hydrolysis.

The hydrolysate was freeze-dried and stored at −20 °C until use.

### 2.12. Determination of Protein Content

The Dumas method (AOAC 992.15) [40] was used to determine the nitrogen content using the conversion factor of 5.55 for nitrogen to protein, using a LECO TruMac Nitrogen analyser from Leco Corp. (AG, Geleen, The Netherlands). The results were expressed per 100 g of dried sample.

### 2.13. DPPH Radical Scavenging Activity

The antioxidant activity of the gelatin hydrolysates was determined according to Mosquera, Gómez, Montero and Giménez (2016) [19]. A solution was prepared by dissolving 3.5 mg of DPPH+ (1,1-diphenyl-2-picrylhydrazyl) in 10 mL of methanol. The solution was then diluted with more methanol until the absorbance at 515 nm was 1 ± 0.009. Next, 1.95 mL of the diluted DPPH+ solution was mixed with 0.05 mL of the hydrolysate (previously diluted to 0.06 g/mL with distilled water). The resulting mixture was stirred and allowed to stand for 30 min at room temperature. After centrifugation at 6000× *g* for 10 min, the absorbance of the supernatant was measured at 515 nm using a spectrophotometer (UV-160 A, Shimadzu, Kyoto, Japan). The results were expressed as percentages.

## 3. Results

### 3.1. Purification of the Enzymatic Extract

**Figure 1:**
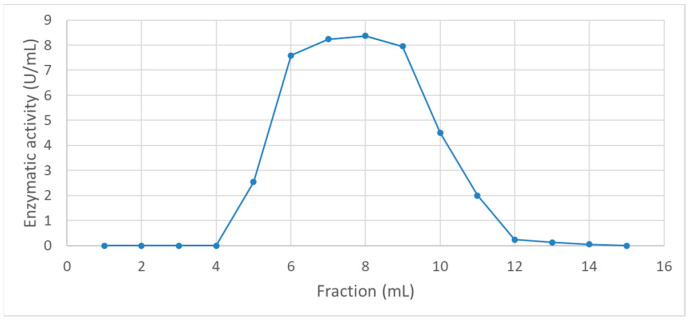
Partial purification curve of the enzymes extracted from *B. subtilis* cultivated using tuna muscle as a substrate.

The partial purification curve of the enzymes extracted from B. subtilis cultivated using tuna muscle as a substrate is shown in Figure 1. Among the collected fractions, the highest enzymatic activity was observed in the eighth fraction, where the activity increased from 6.12 ± 0.093 U/mL (unpurified sample) to 8.37 U/mL. These findings confirm the successful generation of a semi-purified enzymatic extract, achieved through the removal of non-enzymatic residues and enzymes with low activity. The results indicate that a purer enzymatic extract was obtained, entailing the elimination of non-enzymatic residues and low-activity enzymes.

### 3.2. Molecular Size Determination of Enzymatic Extract

**Figure 2:**
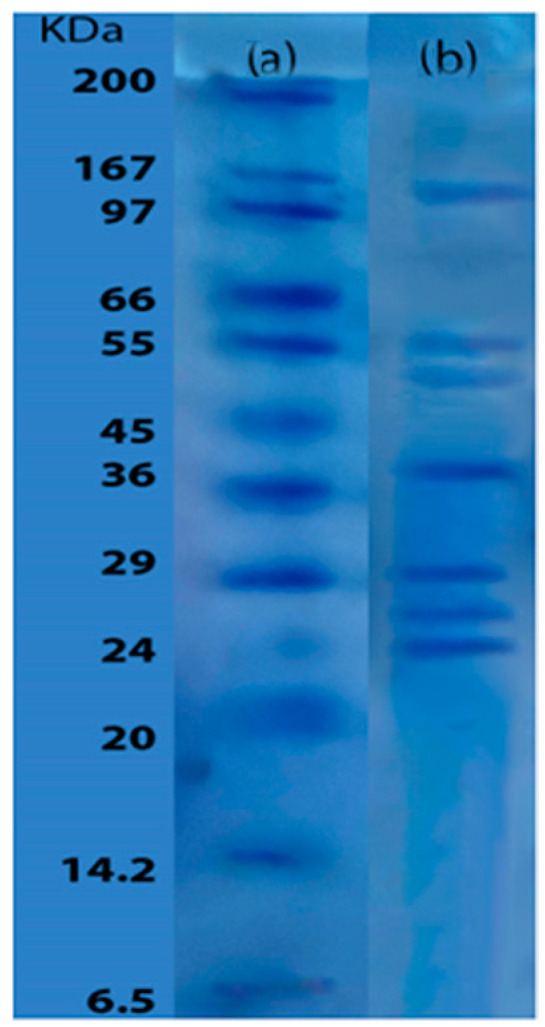
SDS-PAGE gel of enzymatic extract and molecular weight standard. (**a**) Molecular weight standard; (**b**) enzymatic extract.

The molecular weight profile of the enzymatic extract is shown in Figure 2, lane b. Several bands of different molecular sizes were observed, indicating the presence of diverse proteins in the extract. The most abundant component had a molecular weight of approximately 150 kDa, while the smallest one had a molecular weight of approximately 24 kDa, as determined by the reference pattern used (lane a). Notably, the molecular weights of the last three bands were below 30 kDa, while the second and third bands were in the range of 55 to 50 kDa. It is important to consider the possibility that these bands may correspond to enzymes as well as other proteins present in the extract.

### 3.3. Amino Acid Composition

The gelatin obtained had a total protein content of 83.8 ± 0.2% and exhibited an extraction yield of 21 ± 0.3%. The amino acid composition of gelatin is depicted in Table 1. It is noteworthy that glycine emerges as the predominant amino acid, with a remarkable abundance of 20.5%. Furthermore, significant contents of proline and hydroxyproline were detected, with values of 12.1% and 10.3%, respectively.

Furthermore, the presence of alanine (9.8%), arginine (8.6%) and glutamic acid and glutamine (5.3%) in gelatin was relevant. It is noteworthy that the composition and sequence of amino acids in gelatin showed specific variations depending on the species studied. However, the predominant presence of glycine and proline is consistently observed across different species [41]. These results contribute to the characterization of yellowfin tuna gelatin and provide valuable insights for its potential applications in various scientific and technological fields. The amino acid composition plays a crucial role in determining the functional properties of both gelatin and its hydrolysates, especially in terms of antioxidant activity.

### 3.4. Viscoelastic Properties and Gel Strength

**Figure 3:**
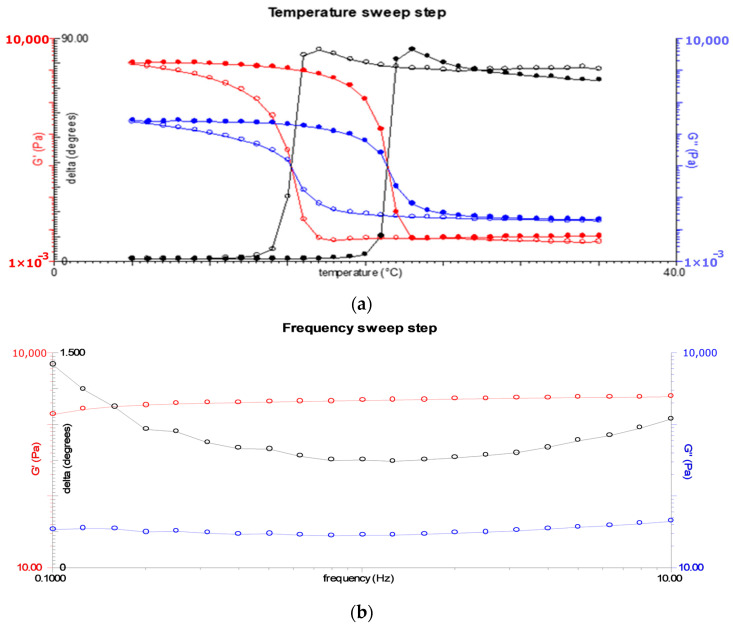
Viscoelastic properties: (**a**) changes in elastic modulus (red), viscous modulus (blue), and phase angle (black) as function of temperature; (**b**) changes as a function of angular frequency in elastic modulus (red) and viscous modulus (blue). The cooling ramp is represented by the open symbols and the heating ramp by the solid symbols.

Figure 3a shows the viscoelastic properties of yellowfin tuna skin-derived gelatin as a function of temperature. Analysis of the phase angle indicated that gelation began at 16 °C, and the onset of gelation was observed at this temperature. In addition, the melting temperature was determined to be 22 °C. It is noteworthy that the gelatin exhibited a higher melting point than the gelation point, suggesting an energy absorption phenomenon during the melting process [42].

**Figure 4:**
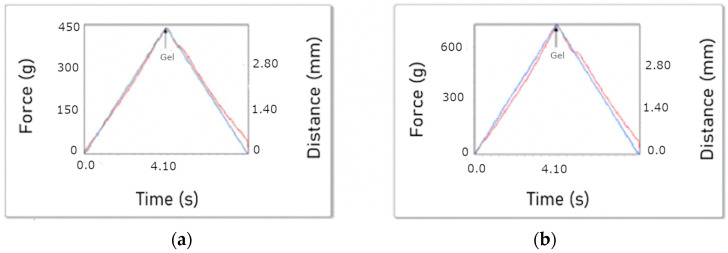
Gel strength: (**a**) yellowfin tuna skin gelatin; (**b**) gelatin coil with 260 °Bloom.

Figure 3b depicts the relationship between the elastic modulus (G’) and the viscous modulus (G”) as a function of angular frequency, offering insights into the rheological behavior of the gelatin gel. In particular, G’ exceeded G”, indicating the formation of a gel. Moreover, the gel strength of the sample was determined to be 425 ± 11 g, as illustrated in Figure 4a. For comparison purposes, the gel strength of a commercial gelatin sample (260 °Bloom) was also determined, yielding a value of 701 ± 7 g (Figure 4b). Based on this comparison, an approximate gel strength of 160 °Bloom can be inferred for the extracted yellowfin tuna gelatin.

### 3.5. Optimization of Gelatin Hydrolysis Conditions

Table 2 presents the degree of hydrolysis as a function of the temperature and enzyme extract concentration, both recognized factors known to influence the extent of proteolysis. According to the experimental design, the maximum degree of hydrolysis was achieved under conditions of 50 °C and 6.5 IU, resulting in a value of 19.81%.

The response surface experimental design predicted a maximum antioxidant activity of 35.70% at 70 °C and 1.5 enzyme units. The analysis of variance yielded a high R-squared value of 98.35%, confirming the reliability of the prediction. The formula derived from the analysis is as follows:

(2) $$Antiox.act.\left(\%\right)=-28.35+2.00\times Temperature-6.51\times enzymeunits-0.014\times {Temperature}^{2}\;+0.048\times Temperature\times enzymeunits+0.23\times {enzymeunits}^{2}$$

**Figure 5:**
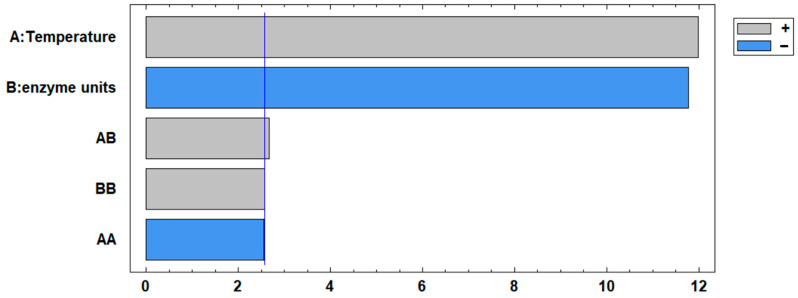
Pareto chart for antioxidant activity: A represents the linear effect of temperature, while B denotes the linear effect of enzyme units. AA indicates the quadratic effect of temperature, BB signifies the quadratic effect of enzyme units, and AB represents the interaction between the two variables.

The results suggest that an increase in temperature causes a decrease in the degree of hydrolysis, possibly due to thermal denaturation of the enzymes. This denaturation affects the tertiary structure and reduces the ability to cleave large proteins into peptides or induces protein aggregation [43]. The lowest degree of hydrolysis was recorded under conditions of 70 °C and 1.5 IU, reaching 12.77%. The decrease in hydrolysis may be due to a lack of catalytic sites [44]. The results also suggest a relationship between a low degree of hydrolysis and a high antioxidant capacity, highlighting the importance of longer peptides in this activity. This is supported by Figure 5, which shows a statistically significant (*p* < 0.05) positive effect on antioxidant activity. Furthermore, it was found that an increase in the enzyme units used had a negative effect on the antioxidant activity, confirming that extensive hydrolysis is not required to produce a potent antioxidant hydrolysate. Moreover, both temperature and enzyme units had a statistically significant quadratic effect (*p* < 0.05). The statistical analysis confirms the evidence of an optimal range for both temperature and enzyme units, beyond which further changes may result in a decrease in activity.

## 4. Discussion

### 4.1. Purification of the Enzymatic Extract

The results obtained in this study (Figure 1) demonstrate the efficacy of the purification process in enhancing the activity and purity of the enzymatic extract. The increase in enzymatic activity in the purified fractions suggests that the method used was effective in concentrating the enzymes while removing unwanted components. The effectiveness of the purification process can be attributed to the use of a combination of ammonium sulfate precipitation and gel filtration chromatography. Specifically, Sephadex G-25 was used for desalting, allowing the removal of low-molecular-weight contaminants and salts, which could interfere with subsequent purification steps. Following this, Sephadex G-100 gel filtration chromatography effectively separated proteins based on their size, facilitating the isolation of the desired enzymes from other proteins and contaminants [45]. These techniques are widely recognized for their ability to concentrate and purify proteins by removing contaminants and separating proteins based on their size and solubility. Similar methodologies have been used in other studies to purify various enzymes, including polyphenol oxidase (PPO) from potatoes and keratinolytic proteases from feather meal bioconversion by *Bacillus* sp. P45 [45]. Another study demonstrated that using ammonium sulfate precipitation followed by affinity chromatography with different spacer arms significantly improved the purification fold and specific activity of PPO [46].

### 4.2. Molecular Size Determination

The electrophoretic profile analysis of the enzymatic extract obtained from yellowfin tuna provided a preliminary insight into the molecular sizes of the detected proteins. Among the observed proteins, the highest molecular weight recorded was 150 kDa, which is greater than the molecular weights reported in the “Brenda” enzyme database for proteases from other *B. subtilis* strains. The electrophoretic profile indicated a band corresponding to a molecular weight around 86.7 kDa, which is similar to thermolysin (EC 3.4.21.53) [47]. Additionally, the electrophoresis showed other bands corresponding to varying molecular sizes, probably corresponding to proteases. For example, a band around 53 kDa may correspond to a C-terminal processing enzyme (EC 3.4.21.102), which is consistent with a similar molecular weight reported for a metalloprotease from *B. stearothermophilus* [47]. Comparison with the “Brenda” enzyme database in NCBI for *B. subtilis* suggested the possible presence of other enzymes in the electrophoretic bands. The fourth band (37 kDa) could correspond to both endopeptidase La (EC 3.4.21.53) and subtilisin (EC 3.4.21.62), aligning with previous studies that identified a metalloprotease (37 kDa) in *B. subtilis* [48]. Additionally, bands with molecular weights of approximately 27 and 29 kDa were observed, which is consistent with previous studies on proteases from *Bacillus* strains, typically ranging from 28 to 70 kDa [49]. Interestingly, an alkaline protease from *B. subtilis* of 27 kDa was identified in a previous study using industrial by-products as a fermentation medium, resembling the molecular weight observed for the penultimate band in this study [50]. Furthermore, a comparable molecular weight of approximately 30 kDa was reported in a study utilizing *B. subtilis* and agricultural residues as substrates [51]. The sixth band could be associated with a fibrinolytic enzyme identified at 29 kDa, which was isolated and characterized in previous research [49]. Additional studies identified an extracellular protease with a molecular weight of 29 kDa, characterized as subtilisin, which could correspond to the sixth band [52].

Finally, the Brenda database provided additional insight, indicating that the repressor LexA enzyme (registered as EC 3.4.21.88) with a molecular weight of 24 kDa could correspond to the smallest-molecular-weight molecule observed in the electrophoretic profile. Additionally, a keratinolytic protease from *B. subtilis* with a molecular weight of 25.4 kDa, similar to the last band in Figure 2, was also found [53]. Although the molecular weights determined by SDS-PAGE provide general protein data, the identified molecular weights, along with the proteolytic activity of the extract, deepen the understanding of the potential of *B. subtilis* for enzyme production using yellowfin tuna muscle as a nitrogen and carbon source in the culture medium.

### 4.3. Amino Acid Composition

As expected, the gelatin had a high glycine content (20.5%). This is because glycine is required in every third position of the protein to achieve a compact packing of the triple helix of the native protein (collagen) [54]. The presence of proline and hydroxyproline contributes significantly to the thermal stabilization of the collagen triple helix and enhances its ordered conformation by forming a gel-like network [55]. The content of imino acids (Pro and Hyp) was found to be 22.4%, which is comparable to the findings of a study on tuna skin gelatin that reported similar values. These values are within the expected range for imino acid content in gelatins derived from warm-blooded animal tissues [56]. Moreover, the tuna gelatin had low levels of methionine, cysteine, and tyrosine, with values of 2.1, 0.5, and 0.6%, respectively, which is consistent with previous research [54].

### 4.4. Gel Strength

The rheological properties of gelatin primarily determine its quality, with the gel strength (measured in Bloom degrees) being a key parameter for evaluation. The main factors influencing the rheological properties of gelatin are the extraction conditions and the characteristics of the raw material, which affect both the amino acid composition and the molecular weight profile [57]. Gelatins that more closely resemble the triple-helix structure upon cooling, resulting in improved rheological properties, are associated with higher average molecular weights and increased content of imino acids (Pro + Hyp) [34,58]. The gelatin obtained in this study had a favorable composition of these imino acids as previously discussed. Gel strength measurements typically use a “Bloom jar” container that requires approximately 155 mL of gelatin solution, which is approximately 10 g of gelatin. However, the use of Bloom jars is not practical for many scientific studies due to the large sample volumes required. As a result, researchers often use alternative containers of different sizes and shapes, leading to significant variation in the results obtained. This lack of standardization makes it difficult to compare data from different studies [35].

To ensure a meaningful comparison, the same procedure was performed using a commercially available bovine gelatin with a Bloom value of 260°. The resulting gel strength was then used to estimate the Bloom strength of the yellowfin tuna gelatin. Based on this comparison, it can be inferred that the yellowfin tuna skin gelatin may have a Bloom strength of approximately 160°. This finding is consistent with a previous study in which the gel strength of tuna skin gelatin was reported to be 191.0° Bloom, which is lower than the commercial gelatins analyzed in the same study [41]. Previous research has shown that yellowfin tuna gelatin tends to have a lower gel strength than commercial gelatins. Gel formation and strength can vary due to factors such as amino acid composition, particularly proline and hydroxyproline, and the methods used for extraction, filtration, and drying [59]. However, a previous study found that yellowfin tuna gelatin had a higher Bloom value (426) than bovine gelatin. This difference may be attributed to the extraction method, which involved alkaline hydrolysis over a period of 5 days [60]. The prolonged exposure to alkaline hydrolysis facilitated the extraction and solubilization of collagen proteins, resulting in gelatin with an 89% of hydroxyproline content. This value is comparable to that observed in mammals, which typically ranges from 90.1% to 90.7%, as reported in the literature [61]. Therefore, research suggests that maximizing gel strength can be achieved by extending the extraction time [60].

The approximate Bloom value obtained for the yellowfin tuna gelatin extracted in the present work is comparable to that of other fish skin gelatins. For example, snapper skin gelatin has a value of 108 [54], unicorn leather jacket (*Aluterus monoceros*) skin gelatin has a value of 150 [62], gray triggerfish (*Balistes capriscus*) skin gelatin has a value of 168.3 [63], and cod skin gelatin has a value of 180 [64]. While the gelatin in the present study has a lower gel strength than bovine gelatin, it is important to note that gelatin with different Bloom strengths serves different commercial applications. High-gel-strength gelatins, such as bovine gelatin (260 g Bloom), are ideal for products that require a firmer texture and higher water retention and are commonly used in the production of molded gelatins, mousses, gummy bears, and marshmallows [20]. Gelatins with lower gel strength, such as those obtained from fish, are more suitable for specific applications in bakery products, food additives, coatings for drug capsules, and technical applications such as the production of gelatin films for coatings in the photographic industry [58]. Additionally, fish gelatin, including that from yellowfin tuna, may benefit certain applications that require lower gel strength, such as soft gels or desserts where a more tender texture is desired. Another advantage of fish gelatin over bovine or porcine gelatins is the absence of religious restrictions (such as those in Hinduism, Islam, Adventism, and others) and the absence of health concerns such as foot-and-mouth disease, bovine spongiform encephalopathy, and transmissible encephalopathy [58].

### 4.5. Viscoelastic Properties

The gelation and melting temperature values obtained in this study fall within the typical range observed for fish skin gelatins, which are generally reported to be between 8 and 25 °C and between 11 and 28 °C, respectively [65]. Previous research showed that gelatin derived from warm-water fish species may have a thermal stability closer to that of mammalian gelatin than that of cold-water species [58]. However, it is imperative to consider variables such as fish species, raw material, and processing conditions, which have a significant impact on thermal stability [66]. These results demonstrate the suitability of the gelatin obtained for food applications.

Previous studies using yellowfin tuna skin gelatin reported gelation and melting points like those found in this research (18.7 °C and 24.3 °C, respectively) [60]. In contrast, other authors reported lower gelation and melting points of tuna skin gelatin (10 °C and 13.4 °C, and 18 °C and 20.6 °C, respectively [67]). In another study, gelatins with melting points ranging from 23.3 °C to 28.4 °C were obtained, concluding that the longer the extraction time, the lower the thermostability (melting point) of the derived gelatins [68]. These variations can be attributed to the different extraction conditions and tuna species, resulting in different molecular weight profiles and amino acid compositions [69]. The gelling properties of gelatin are known to be positively influenced by the proportion of alpha and beta chains, whereas they are negatively affected by the presence of low-molecular-weight fragments [70].

### 4.6. Degree of Hydrolysis

The degree of hydrolysis (DH) plays a crucial role in determining the extent of protein fractionation, resulting in the release of peptides with various biological activities such as antioxidant, antihypertensive, anti-inflammatory, nootropic, hypoglycemic, antimicrobial, and antiproliferative, among others [71]. Under the experimental conditions of this study (temperatures ranging from 55 to 65 °C and enzyme concentrations ranging from 1.5 to 6.5 IU), the highest degree of hydrolysis obtained was 19.81%.

In a previous study, yellowfin tuna meat was used as raw material to produce a hydrolysate with emulsifying and foaming properties using papain, resulting in a DH of 14.35% [72]. This relatively low DH resulted in the prevalence of components above 10 kDa (60%) and may be related to the substrate specificity of papain, which may be different from the enzyme used in the present research. Conversely, another study investigated the effects of different enzymes and hydrolysis times using tuna skin gelatin as the raw material, resulting in DHs ranging from 76% to 85% [73]. Notably, a treatment time of approximately 3 h showed the highest efficiency, which is consistent with previous findings suggesting that a stationary phase, characteristic of protease-induced protein hydrolysis, is reached around this time. In a separate study, yellowfin tuna skin gelatin subjected to hydrolysis with 2% alcalase showed a significantly higher DH of 45.29% [59]. The discrepancy between the DH obtained in that study and the present study (15–24%) can be attributed to the high proteolytic activity of alcalase, resulting in more extensive protein breakdown. Furthermore, hydrolysis of flounder skin gelatin with alcalase for 2 h yielded DH values of 23.6%, similar to those obtained in this study. The variation in DH can be attributed not only to differences in hydrolysis conditions and enzyme concentration, but also to variations in enzymatic affinity for different substrates [74]. The degree of hydrolysis achieved during protein hydrolysis is crucial as it directly influences the biological activities of the resulting peptides.

### 4.7. Antioxidant Activity

An inverse relationship between DH and antioxidant activity was observed: as DH decreases, antioxidant activity increases, with the highest value being 35.96 ± 0.22%. Longer peptides resulting from limited hydrolysis may retain specific secondary structures important for antioxidant activity [74]. Loss of these structures due to more extensive hydrolysis could reduce antioxidant potency. The radical scavenging capacity of hydrolysates depends on several factors, including peptide size, peptide sequence, amino acid composition, enzyme used, and degree of hydrolysis. Among these factors, both amino acid composition and peptide sequence play a crucial role in defining the antioxidant potency [74]. 

Peptides generated through limited hydrolysis, which involves breaking down protein molecules into shorter peptide chains without completely degrading the protein, have enhanced electron and hydrogen donating capabilities, allowing them to effectively scavenge free radicals [75]. It is important to note that the use of *B. subtilis* enzymatic extract, by affecting the size of peptides, also influences their sequence and, ultimately, the antioxidant activity of the resulting hydrolysates [59]. That is, the hydrolytic capacity of this enzymatic extract results in the generation of peptides with antioxidant properties. Furthermore, these results are consistent with a previous study in which hydrolysates obtained after 3 h of hydrolysis showed superior antioxidant activity compared to those obtained using extended hydrolysis times (4 and 5 h). This suggests the existence of an optimal hydrolysis time, beyond which the antioxidant activity may decrease due to the degradation of high-MW peptides [76]. In addition to DH, the presence of specific residues within the peptide sequence may contribute to their antioxidant potential. Amino acids such as glutamic acid, glutamine, and arginine have been associated with increased antioxidant activity [77]. In the context of the present study, the gelatin obtained was found to have a significant content of these amino acids. The amino acid composition was particularly enriched in arginine (8.6%) and glutamic acid + glutamine (10.2%). In previous research, the gelatin extracted from yellowfin tuna skin showed an amino acid composition particularly enriched in arginine (9.16%) and glutamic acid (10.31%), demonstrating excellent antioxidant activity [59]. Similarly, in another study, arginine and glutamic acid were measured at levels of 7.06% and 8.81%, respectively [55]. In another study focusing on the amino acid composition of yellowfin tuna skin gelatin, it was found that after the prominent presence of glycine, proline, and hydroxyproline, the levels of arginine and glutamic acid were found to be significant [73]. The hydroxylated amino acids hydroxyproline (10.3%) and hydroxylysine (1%) were also found in this study. These amino acids are known to confer additional antioxidant properties. Hydroxyproline enhances the stability of the triple-helix structure of collagen, which plays a crucial role in the antioxidant activity of gelatin-derived peptides. These amino acids provide additional stability and enhance the radical scavenging capacity of the peptides. Hydroxyproline and hydroxylysine are predominantly found in collagen and its derivatives, such as gelatin, and are typically present in lower amounts in other protein sources [21].

In a recent study, the antioxidant activity of a yellowfin tuna skin hydrolysate obtained using alcalase was investigated, and high scavenging rates around 90% were found [41]. The efficacy in DPPH radical scavenging activity was lower when the enzyme used was neutrase (51.8%) and flavourzyme (44.1%). These values are higher than those obtained in the present study (35.96%). The differences are attributed to the type of enzyme used, with different enzymatic cleavage sites. However, when comparing the results obtained with a previous study in which alcalase was used, a similar scavenging effect (33%) was observed [73]. This highlights the potential of yellowfin tuna residues as a valuable source of antioxidants. Similarly, a protein hydrolysate obtained from by-products of bigeye tuna (*T. obesus*) showed remarkable antioxidant activity, reaching 57.08% [78]. In another study, the DPPH radical scavenging activity of hydrolyzed collagen from yellowfin tuna (*T. albacares*) skin ranged from 36.54% to 69.39% [77]. An additional study demonstrated the release of antioxidant peptides from protein of the same tuna species using papain, showing an activity level of 56.74% [75]. It is noteworthy that the studies mentioned above used commercially available enzymes; the values obtained in this study were obtained using enzymes extracted directly from *B. subtilis* using tuna muscle tissue as a growth medium. This highlights the remarkable antioxidant activity that can be achieved by this method, which is characterized by its economic viability and ease of acquisition. Importantly, the results obtained are in close agreement with those reported in previous research, as discussed above. In addition, various by-products of marine species, including round scad (*D. maruadsi*) [79], red snapper (*L. vitta*) [80], skipjack tuna (*K. pelamis*) [81], and yellowstripe scad (*S. leptolepis*) [82], have been investigated for the release of antioxidant peptides by protein hydrolysis. These studies have highlighted the potential of such by-products to act as electron donors for free radicals. Therefore, the exploration of low-value marine products, particularly the peptide fractions of collagen by-products from the seafood industry, offers a vast and promising field of research.

## 5. Conclusions

In conclusion, this study demonstrates the potential to valorize by-products derived from the industrial processing of yellowfin tuna, specifically the tails. The muscle protein can be used as a source of nitrogen and carbon in a growth medium for *Bacillus subtilis*. This strain produces proteases that hydrolyze the gelatin from the skin, releasing antioxidant peptides. Additionally, the unhydrolyzed gelatin exhibits excellent rheological properties and could have numerous applications in diverse fields such as food and cosmetics. The results highlight the effectiveness and potential applicability of this methodology for the valorization of these by-products, contributing to their sustainable use. The next phase of the study will focus on the valorization of other industrial by-products from yellowfin tuna processing, including tail bones (a source of collagen) and viscera (a source of proteases). Moreover, the bioactive potential of the protein hydrolysates obtained in this work, including their antimicrobial, antihypertensive, hypoglycemic, and antiproliferative properties, should be evaluated. The results of these studies will provide a comprehensive understanding of the potential of industrial yellowfin tuna by-products, which currently lack commercial value, as sources of high-value ingredients.

## Figures and Tables

**Table 1:** Amino acid composition of gelatin from yellowfin tuna skin.

Amino Acids	g/100 g Amino Acids (%)
Asp + Asn	5.33
Thr	3.23
Ser	3.69
Glu + Gln	10.16
Pro	12.13
Gly	20.50
Ala	9.81
Cys	0.49
Val	1.81
Met	2.09
Ile	0.91
Leu	2.59
Tyr	0.60
Phe	2.31
His	0.75
Lys	3.66
Arg	8.63
OH-Pro (Hyp)	10.31
OH-Lys	1.00
Total	100

**Table 2:** Hydrolysis degree and antioxidant activity of the hydrolysates of gelatin from yellowfin tuna at different temperatures and enzyme Units.

Temperature (°C)	Enzymatic Units (IU)	Hydrolysis Degree (% DH)	Antioxidant Activity (%)
70	6.5	16.66	28.65 ± 0.16
70	4	15.40	31.71 ± 0.41
60	4	16.06	28.01 ± 0.49
70	1.5	12.77	35.96 ± 0.22
60	6.5	18.30	26.60 ± 0.55
60	4	16.24	28.15 ± 0.45
60	1.5	13.46	33.26 ± 0.32
50	4	17.16	22.32 ± 0.54
60	4	16.10	27.65 ± 0.88
50	6.5	19.81	17.64 ± 0.38
50	1.5	16.35	29.77 ± 0.22

## Data Availability

The original contributions presented in the study are included in the article, further inquiries can be directed to the corresponding author/s.
